# American Medical Association

**Published:** 1889-05

**Authors:** 


AMERICAN MEDICAL ASSOCIATION.—The fortieth annual meeting of the Association will be held in Newport R. I. on the 25th of June next.

The Chairman of Committees is Dr. R. H. Stover and the local Sect'y. is Dr. V. M. Frances, who will impart information in regard to section work and programme. As there are 13 Hotels in the City of Newport, it is inferred that the entertainment will be cheap and abundant.

